# The Large-Conductance, Calcium-Activated Potassium Channel: A Big Key Regulator of Cell Physiology

**DOI:** 10.3389/fphys.2021.750615

**Published:** 2021-10-21

**Authors:** Maria Sancho, Barry D. Kyle

**Affiliations:** ^1^Department of Pharmacology, University of Vermont, Burlington, VT, United States; ^2^Department of Pathology and Laboratory Medicine, College of Medicine, University of Saskatchewan, Saskatoon, SK, Canada

**Keywords:** BK channels, smooth muscle, nervous system, membrane potential, intracellular Ca^2+^

## Abstract

Large-conductance Ca^2+^-activated K^+^ channels facilitate the efflux of K^+^ ions from a variety of cells and tissues following channel activation. It is now recognized that BK channels undergo a wide range of pre- and post-translational modifications that can dramatically alter their properties and function. This has downstream consequences in affecting cell and tissue excitability, and therefore, function. While finding the “silver bullet” in terms of clinical therapy has remained elusive, ongoing research is providing an impressive range of viable candidate proteins and mechanisms that associate with and modulate BK channel activity, respectively. Here, we provide the hallmarks of BK channel structure and function generally, and discuss important milestones in the efforts to further elucidate the diverse properties of BK channels in its many forms.

## Introduction

Large-conductance Ca^2+^-activated K^+^ or BK [Big Potassium (K^+^)] channels, also known as Maxi-K, Slo1 or K_Ca_1.1 channels, are ubiquitously expressed in a broad array of excitable and non-excitable cells including neurons/glial cells (Trimmer, 2015; Hayashi et al., 2016; Latorre et al., 2017), a variety of vascular or nonvascular smooth muscle (Nelson et al., 1995; Nelson and Quayle, 1995; Brenner et al., 2005; Herrera et al., 2005; Hu and Zhang, 2012; Kyle et al., 2013; Krishnamoorthy-Natarajan and Koide, 2016; Dopico et al., 2018), skeletal muscle (Pallotta et al., 1981), neuroendocrine cells (Solaro et al., 1995) and, epithelial cells (Manzanares et al., 2011; Yang et al., 2017). These channels are characterized by exhibiting a high K^+^ selectivity, a large single channel conductance of 200–300pS (~10–20-fold greater that other K^+^ channels), and an exquisite ability to be dually activated by two distinct physiological stimuli: membrane depolarization and local increases in intracellular Ca^2+^ (Marty, 1981; Pallotta et al., 1981; Barrett et al., 1982; Latorre et al., 1982, 1989; Marty, 1989). Given their unusual and very impressive large unitary conductance, the stimulation of BK channels leads to a rapid efflux of K^+^, which results in membrane hyperpolarization. This capability therefore confers an important physiological mechanism to modulate membrane excitability and intracellular Ca^2+^ homeostasis. Thus, BK channels are key players in a plethora of physiological processes such as smooth muscle contraction (Brayden and Nelson, 1992; Nelson et al., 1995; Pérez et al., 1999; Wellman and Nelson, 2003), neuronal signaling (Robitaille and Charlton, 1992; Hu et al., 2001; Raffaeli et al., 2004; Wang, 2008), hormone secretion (Braun et al., 2008) and audition (Miranda-Rottmann et al., 2010). In the brain, astrocyte endfeet express functional BK channels with the ability to sense astrocytic Ca^2+^. This in turn signals to neighboring arteriolar smooth muscle cells by the focal release of K^+^ into the perivascular space, thus playing an essential role in neurovascular coupling and the regulation of brain blood flow (Filosa et al., 2006).

In harmony with these vital physiological functions, both malfunctioning and abnormal expression (loss or gain of function) of BK channels can have detrimental consequences on the excitability of neuronal or vascular networks (N’Gouemo, 2014; Contet et al., 2016). Thus, BK channels are now recognized to play a role in numerous pathophysiological conditions including seizures and epilepsy (Du et al., 2005; N’Gouemo, 2011), movement disorders (Du et al., 2005; Imlach et al., 2008), autism and mental retardation (Laumonnier et al., 2006), cerebral ischemia and hypoxia (Gribkoff et al., 2001; Kumar, 2007; Liao et al., 2010; Tao et al., 2015), hypertension (Brenner et al., 2000; Pabbidi and Roman, 2017), obesity (Jiao et al., 2011) and diabetes mellitus (Gutterman and Durand, 2014). In this review, we provide an overview of the basic biophysical features, including structural, functional and pharmacological properties of mammalian BK channels, with a particular focus on their pathological implication as well as their potential as molecular targets for the development of innovative and promising therapeutic strategies in the nervous and cardiovascular systems.

## Biophysical Features of Large-Conductance Ca^2+^-Activated K^+^ Channels

### The Structure of BK Channels

**Figure 1:**
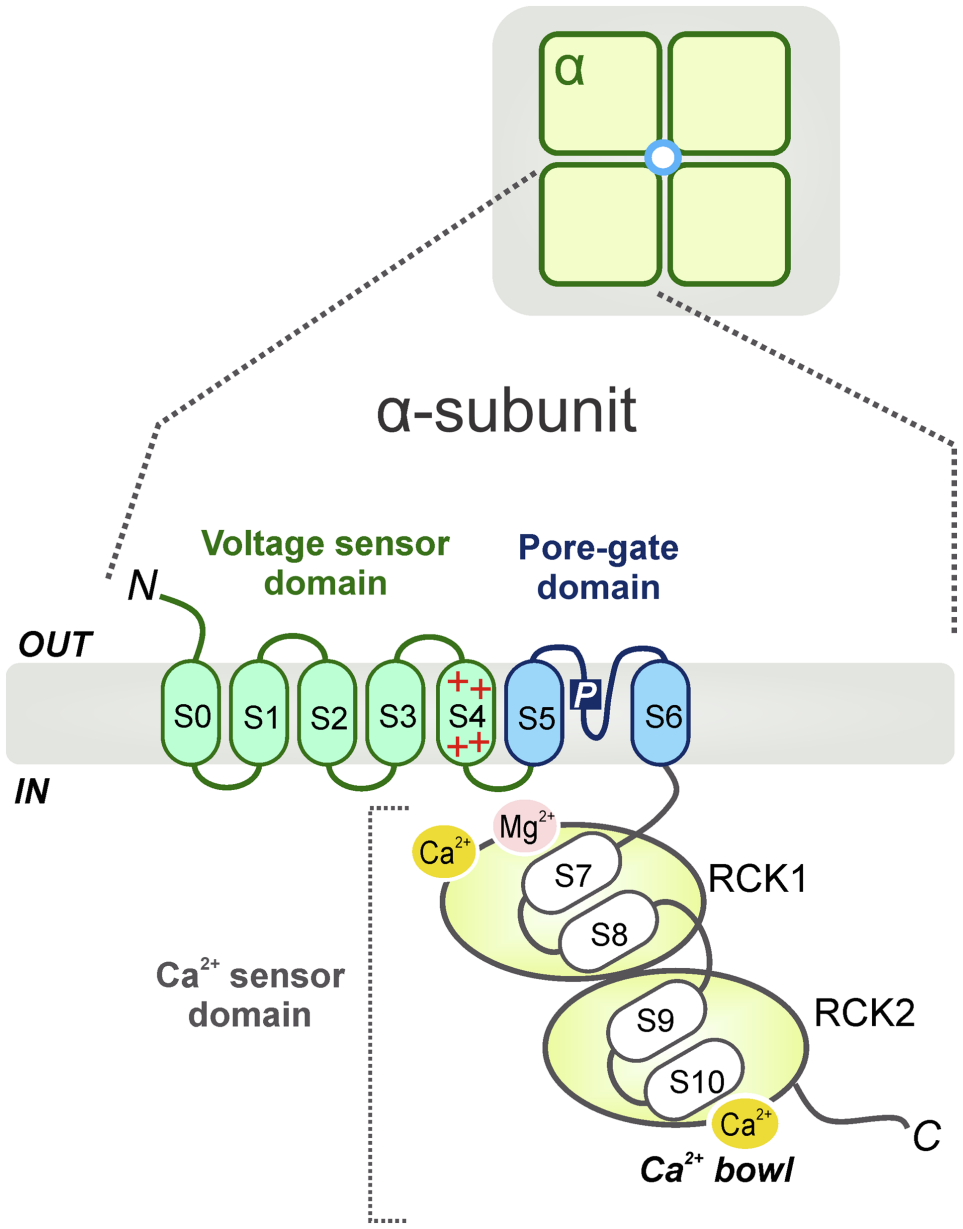
Schematic diagram of the general BK channel structure. BK channels constitute tetramers of the channel pore-forming α-subunits (*top*). Each α (*Slo1*) subunit contains 3 main domains: a voltage sensor domain (VSD, S0-S4), a pore-gate domain (S5-S6) and a C-terminal cytosolic region, which functions as a Ca^2+^ sensor domain (S7-S10) (*bottom*). The Ca^2+^ sensor domain is constituted by two non-identical domains (i.e., RCK1 and RCK2) which contain high-affinity binding Ca^2+^ sites (Ca^2+^ *bowl*) and several modulatory domains for multiple ligands or cations including Mg^2+^.

As members of the TM6 voltage-gated ion channel superfamily, BK channels share partial topology with voltage-gated K^+^ (K_v_) channels and, constitute tetramers of the pore-forming α-subunits or Slo1 proteins, encoded by a single gene, termed *Slo1* or *KCNMA1* in mammals. The *Slo1* gene undergoes extensive alternative splicing (Tseng-Crank et al., 1994; Navaratnam et al., 1997), giving rise to a high degree of functional diversity in BK channels. It was firstly identified in the *Slowpoke* mutant of *Drosophila melanogaster*. This mutant exhibited abnormal locomotor patterns and obvious impaired flight ability due to a deficiency in a Ca^2+^-activated conductance (Elkins et al., 1986; Atkinson et al., 1991). Each α-subunit, containing about 1,200 amino acids, is comprised of 7 membrane-spanning domains (i.e., S0-S6; ~330 amino acids) (Wallner et al., 1996) with S4 considered as a conserved positively charged domain that acts as a well-defined voltage sensor as seen in K_v_ channels (Liman et al., 1991; Lopez et al., 1991; Logothetis et al., 1993; Seoh et al., 1996). The pore-gated domain is constituted by S5 and S6 transmembrane segments; it forms the center of the BK channel and acts as a K^+^ selective filter. Additionally, the α-subunit contains an extensive (~840 amino acids) C-terminal cytosolic region with four additional hydrophobic segments (S7–S10) containing two non-identical domains (RCK1 and RCK2), serving as regulators for K^+^ conductance. Each of the RCK domains contain a high-affinity binding Ca^2+^ site and multiple regulatory domains for a variety of ligands (Jiang et al., 2002; Xia et al., 2002; Sweet and Cox, 2009) or divalent cations including Mg^2+^ (Shi et al., 2002; Xia et al., 2002; Yang et al., 2008; Figure 1). Interestingly, the “gating ring” of the tetramer is constituted by these four RCK1-RCK2 arrangements (Tao et al., 2017). While Ca^2+^ ions are powerful promoters of BK channel open probability, other Ca^2+^-activated K^+^ channel species, activated by lower intracellular Ca^2+^ concentrations [i.e., small (SK) or intermediate (IK) K^+^ channels], display a completely dissimilar channel gating mechanism. This process requires calmodulin (CaM), a small but highly conserved Ca^2+^-modulating protein, to bind with Ca^2+^ ions (Fanger et al., 1999; Adelman, 2016). Specifically, four CaM molecules attach to the channel tetramer causing a conformational change of the S4-S5 linker that promotes the opening of the channel pore in a cooperative manner with high Ca^2+^ sensitivity (Xia et al., 1998).

The structural features of the α-subunit confer unique biophysical properties to the channel including ion permeation, gating and regulation by diverse ligands and intracellular molecules and ions. Notably, the pharmacology and functional features of BK channels, such as their sensitivity to voltage and intracellular Ca^2+^, are prominently impacted by their association with auxiliary and non-pore-forming modulatory β (β_1-4_) (Brenner et al., 2000; Yan and Aldrich, 2010), γ (γ_1-4_) (Yan and Aldrich, 2010, 2012), and LINGO1 subunits (Dudem et al., 2020). Most importantly, the co-assembling with diverse auxiliary subunits gives rise to the existence of distinct BK channel phenotypes with varied functionality, thus increasing channel heterogeneity. Specifically, the identity of the regulatory subunits that associate with BK channels dictates important electrophysiological and kinetic features of the channel including the voltage range of activation, inactivation or deactivation, single-channel current rectification characteristics, as well as pharmacological sensitivity to different channel blockers (Gonzalez-Perez and Lingle, 2019). In this aspect, recent literature has centered on BK channel gating modulation by these auxiliary subunits (Contreras et al., 2012; Torres et al., 2014; Zhang and Yan, 2014), with a particular emphasis on the unique role of the β-1 subunit in BK channels in smooth muscle and kidney (Krishnamoorthy-Natarajan and Koide, 2016; Latorre et al., 2017).

The complexity of BK channel function reflects the intricacy of its protein structure. In the last decade, several studies employing electron cryomicroscopy (cryo-EM) and X-ray crystallographic analysis have begun to provide key structural and biophysical insights into the BK channel gating (Wang and Sigworth, 2009; Lee and Cui, 2010; Yuan et al., 2010; Hite et al., 2017). However, a complete knowledge of BK channel structure is needed to provide not only a more informed understanding of its biological role(s), but also refined targeting in the practical search for novel drugs and compounds to treat diverse BK-associated pathologies while mitigating potential side effects. Importantly, more extensive crystallographic analyses of the diverse auxiliary subunits may add new dimensions to BK channel modulation and add potential targeting options to the channel in a more tissue- or cell-specific manner. This section will review the organization and structural basis for gating the BK channel as they are currently understood.

### The Voltage Sensor and Activation of BK Channels by Membrane Voltage

One of the most defining hallmarks of BK channels is their mechanism of activation by membrane depolarization and changes in cytosolic Ca^2+^ levels in a synergetic fashion. This idea is supported by various allosteric models revealing that BK channels can open the pore gates and allow K^+^ efflux in the absence of voltage sensor activation and Ca^2+^ binding with an intrinsic open probability (P_o_) of ~10^−7^. (Horrigan et al., 1999). These models further demonstrated that in the practical absence of intracellular Ca^2+^ [(Ca^2+^) < 1nM], membrane depolarization is sufficient to reduce the free energy necessary to maximally stimulate voltage-dependent macroscopic ionic currents through BK channels (Cui et al., 1997; Horrigan et al., 1999; Horrigan and Aldrich, 1999). In this sense, a depolarization greater than +200mV was necessary to promote channel opening at ~0.5nM intracellular Ca^2+^. This Ca^2+^-independent activation of the BK channel is reinforced by the fact that the time constant of current stimulation was three orders of magnitude faster than the averaged diffusion-limited time it would take Ca^2+^ to bind the channel during depolarization (Cui et al., 1997). These latter observations combined with their structural similarity to K_v_ channels, suggest that BK channels possess a virtuously voltage-dependent mechanism of gating conferred by the existence of an intrinsic voltage sensor domain (VSD, S0-S4).

Structurally, the VSD in BK channels resembles the voltage-sensing apparatus of K_v_ channels but with an additional N-terminal transmembrane segment (S0), which exhibits similar voltage dependence to the positively charged S4 domain [i.e., contains three Arginine [Arg] residues] and is important for β-subunit modulation (Meera et al., 1997). Diverse electrophysiological assays have demonstrated that the S0 helix is remarkably close to the extracellular domains of S3 and S4 (Liu et al., 2008) and functionally modulates the transition between the resting and the fully active state of the VSD upon membrane depolarization (Koval et al., 2007).

In terms of functionality, the voltage sensor of BK channels differs from that of K_v_ channels in two main aspects. First, the number of voltage-sensing charges (gating charge) of BK channels – measured as the depolarization-induced movement of the VSD – are smaller compared to Kv channels (0.6*e* vs. ~12–13*e* effective gating charges in BK vs. K_v_ channels), suggesting the requirement of more membrane depolarization to move the VSD of BK channels into the fully activated state. This relatively weak voltage dependence allows BK channels to function throughout a wide range of membrane potentials. Second, while in K_v_ channels each of the first four most extracellular charges (Arg residues) of S4 is voltage sensing and contributes to the gating currents (Bezanilla, 2008), only one Arginine residue influences the actual BK channel gating. This observation suggests the existence of additional charged residues outside of S4 with essential contributions to the total amount of gating charges (Ma et al., 2006). Specifically, two supplementary voltage-sensing charged residues (accounting for at least 50% of gating currents) have been described in the BK S2 segment, and the S3 domain carries an additional residue, also involved in charge-related movements of the gating current. Given the various remarkable dissimilarities, it is possible that the BK channels perform a similar but unique mode of structural rearrangement of the VSD during channel gating (Ma et al., 2006). Further studies using the voltage-clamp fluorometry technique have tracked the relative motions of the BK channel VSD domain upon membrane depolarization, providing a fundamental structural basis to gain a better understanding of the voltage-sensing operation of BK channels (Pantazis et al., 2010; Pantazis and Olcese, 2012).

### The Calcium Sensor and Calcium Sensitivity of BK Channels

Ca^2+^ binding promotes BK channel opening independently of the voltage-sensing apparatus of the channel. This evidence was elegantly demonstrated by classic electrophysiology studies in which the steady-state open probability of BK channels increased as the intracellular Ca^2+^ concentration was elevated (from <10nM to 1,000μM) at a fixed transmembrane voltage (Markwardt and Isenberg, 1992) or at very negative voltages (less than −80mV) where voltage sensors are largely in resting states (Horrigan and Aldrich, 2002).

Physiologically, BK channel activity can be enhanced by elevating cytosolic Ca^2+^ concentrations, since Ca^2+^ binds with high affinity to the cytoplasmic domain(s). Several divalent cations (including Ca^2+^) are sensed by the so-called “gating ring,” a large tetrameric arrangement made up of two different regulators of conductance of potassium (i.e., RCK1 and RCK2) domains. As stated above, each RCK domain possesses a high-affinity Ca^2+^ site (commonly known as the “Ca^2+^ bowl”) where 3 Ca^2+^ ions can bind (i.e., 24 Ca^2+^ ions/channel), leading to a change in the conformation of the gating ring and switching into a conducting state (Pau et al., 2011). In addition, BK channel subunits contain at least a low-affinity site for Ca^2+^ and Mg^2+^ at the interface between the voltage sensor and the RCK1 domain (Zhang et al., 2001). Interestingly, when Mg^2+^ ions bind to this site, a charged residue in the S4 domain is repelled, therefore enabling the active configuration of the VSD, and indirectly promoting BK channel opening (Hu et al., 2003).

In addition to Ca^2+^ and Mg^2+^, RCK domains can sense other divalent cations such as Cd^2+^, Ba^2+^, Mn^2+^, Co^2+^, or Ni^2+^, with relatively low selectivity to that for Ca^2+^ (Xia et al., 2002: Zeng et al., 2005). Furthermore, an additional low-affinity-Ca^2+^ binding site for the ions with smaller radii (i.e., Mn^2+^, Co^2+^, Mg^2+^ and Ni^2+^) has been noted by Zeng et al. (2005).

The binding of intracellular Ca^2+^ (or other divalent cations) promotes a leftward shift of the steady-state open probability of BK channels, a process which is correlated with a slowing deactivation of the channel (Barrett et al., 1982). Electrophysiological studies combined with Ca^2+^-dependent kinetic analysis of the BK channel have determined that its Ca^2+^ affinity resides mainly in the low micromolar range (i.e., 1–10μM) (Cox et al., 1997; Contreras et al., 2013), and can be modulated by a variety of ligands and metabolic states (Xia et al., 2002). The spatial interaction between BK channels and voltage-gated calcium channels (VGCC) appears to be critical for promoting BK channel activation at low membrane voltages. In fact, several studies have shown colocalization or the existence of BK-VGCC macromolecular complexes which mediate rapid and focalized Ca^2+^-activated K^+^ signaling in neurons (Berkefeld et al., 2006).

**Figure 2:**
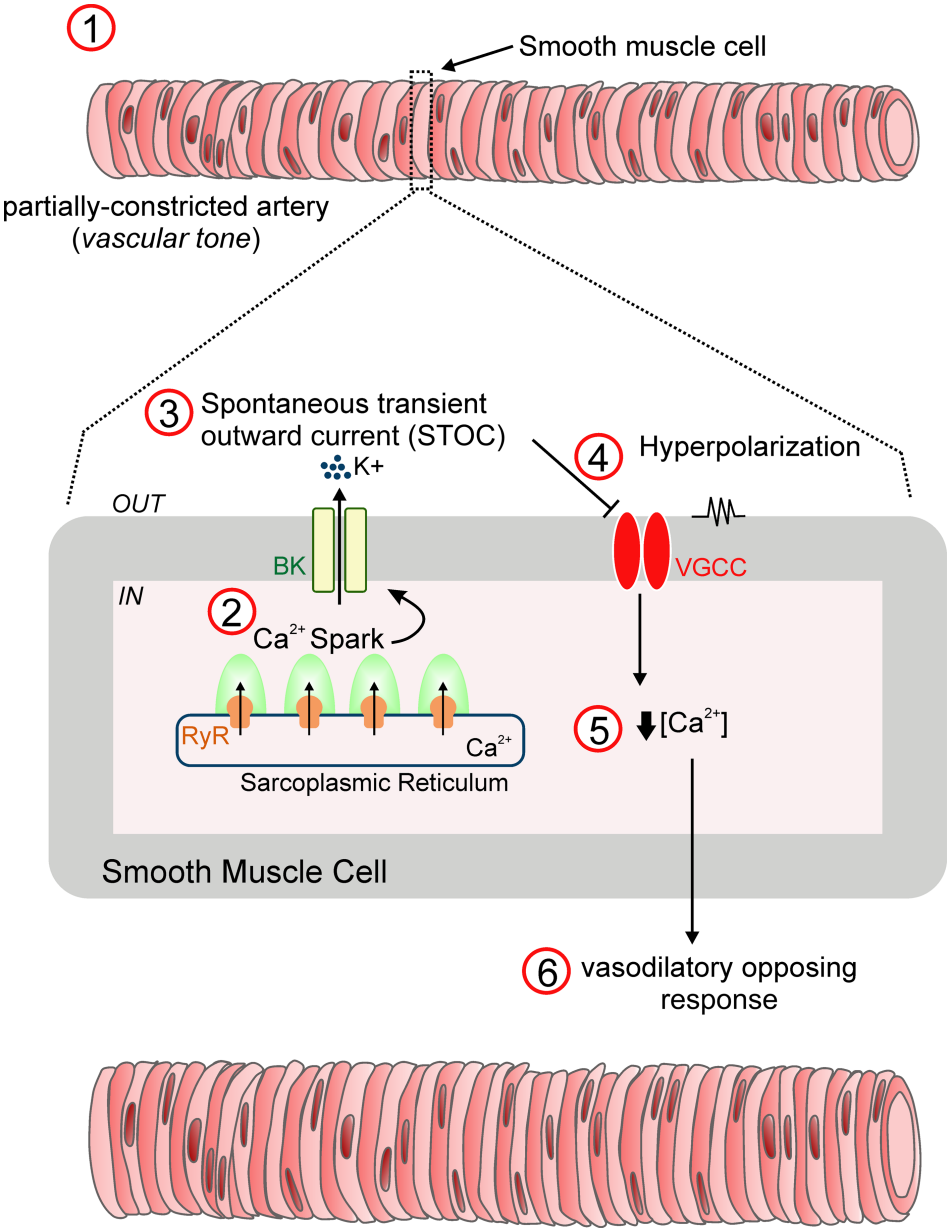
STOC-mediated vasodilation mechanism. In vascular smooth muscle, BK channels are key drivers of negative feedback control *via* the regulation of membrane excitability, an essential mechanism that prevents excessive constriction of resistance arteries (1). Specifically, transient activation of ryanodine receptors (RyR) residing in the sarcoplasmic reticulum leads to the generation of “Ca^2+^ sparks” (2). Single sparks increase the Ca^2+^ concentration in the vicinity of membrane BK channels, provoking their opening and the subsequent development of macroscopic K^+^ currents referred to as “Spontaneous transient outward currents (STOCs)” (3). This in turn, contributes to membrane hyperpolarization by reducing the voltage-gated Ca^2+^ channel (VGCC) open probability (4), and a relative reduction in the intracellular Ca^2+^ levels (5). As a result, the resistance artery develops a dilatory response (6), a vital feedback mechanism to optimize arterial tone development (Nelson et al., 1995).

In vascular smooth muscle, BK channels are associated with elevations in intracellular Ca^2+^ concentrations by behaving as a negative feedback mechanism to oppose the nearly always partially constricted state of resistance arteries (i.e., vascular tone) exhibited under physiological conditions (Figure 2). In particular, highly localized intracellular Ca^2+^ transients, known as “Ca^2+^ *sparks,”* are triggered by the concurrent opening of a number of ryanodine-sensitive Ca^2+^ release (RyR) channels in the sarcoplasmic reticulum. This in turn elevates local Ca^2+^ levels (10–100μM) that activates multiple adjacent BK channels, leading to transient macroscopic currents referred to as spontaneous transient outward currents (i.e., “STOCs”) and subsequent membrane hyperpolarization by closing VGCC and decreasing intracellular Ca^2+^ concentrations (Nelson et al., 1995; Bonev et al., 1997; Porter et al., 1998). Notably, simultaneous recordings of Ca^2+^ sparks and cell membrane potential revealed that Ca^2+^ sparks elicited up to ~20mV hyperpolarization of arterial smooth muscle through the activation of BK channels (Ganitkevich and Isenberg, 1990). Accordingly, blockade of either Ca^2+^ sparks or BK channels depolarized pressurized cerebral arteries causing an increase in intracellular Ca^2+^ levels and subsequent vasoconstriction (Nelson et al., 1995; Knot et al., 1998; Porter et al., 1998). These relevant studies provided solid evidence for Ca^2+^ sparks functioning as essential regulators of the vascular tone through the activation of BK channels.

## Pharmacology of BK Channels

**Figure 3:**
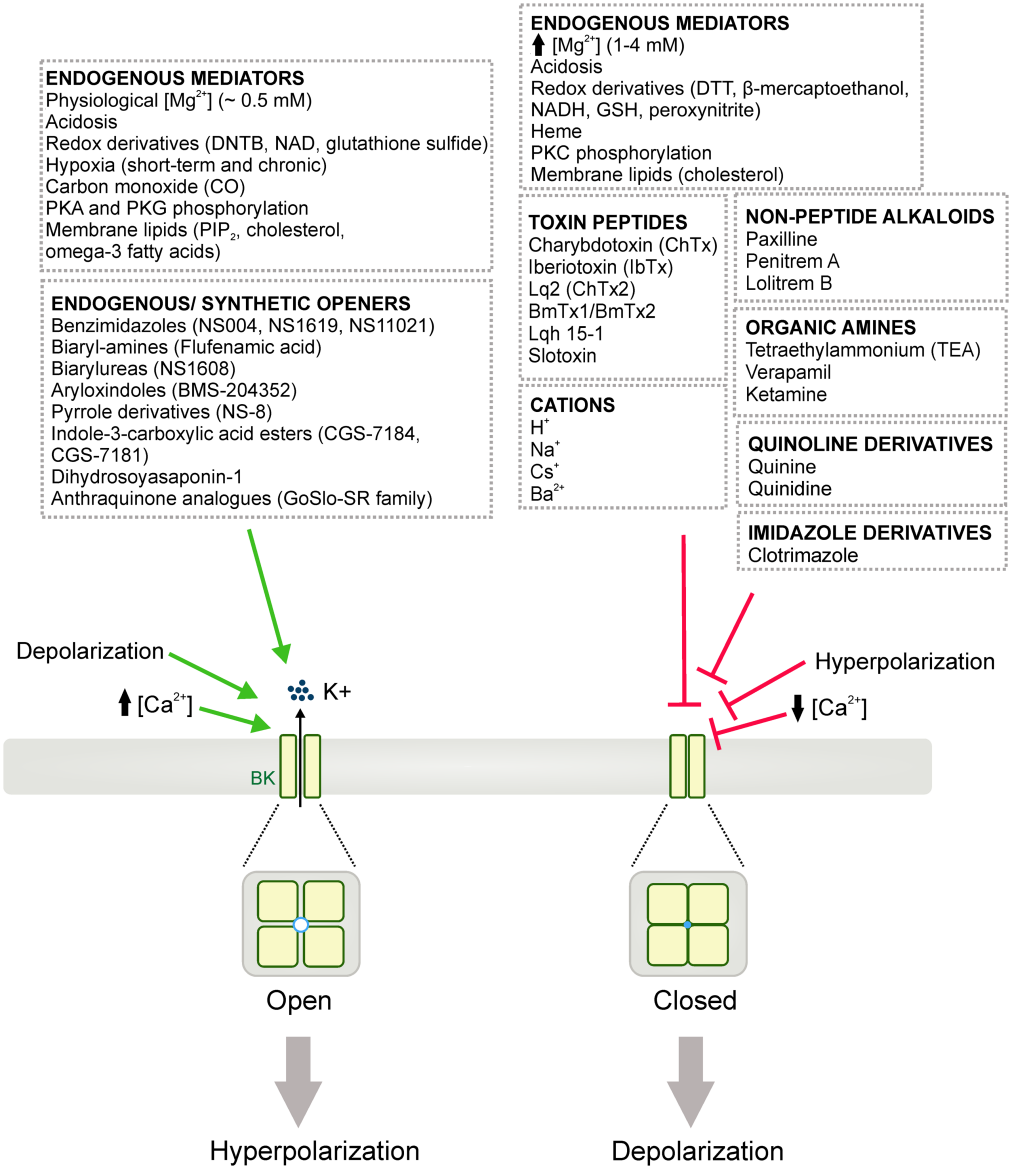
Diagrammatic summary of the pharmacology of BK channels. BK channels can be activated (i.e., opened) or blocked (i.e., closed or inhibited) leading to cell membrane hyperpolarization and depolarization, respectively. Diverse endogenous mediators, redox derivatives and, signaling proteins are able to either potentiate or inhibit BK channel activity. Numerous BK channel inhibitors/blockers have been also reported, including: toxin peptides from scorpion venoms, non-peptide alkaloids, organic amines, quinolone and imidazole derivatives. Additionally, an extensive list of cations including H^+^, Na^2+^, Cs^+^ and Ba^2+^ are shown to influence BK channel activity. Similarly, endogenous and synthetic openers have been widely studied as experimental tools and potential therapeutic approaches for different vascular or neurological disorders involving BK channels.

BK channel activity can be modulated by numerous endogenous mediators, intracellular signaling proteins, peptide toxins, small-molecule blockers, and/or endogenous or synthetic openers (Figure 3). This section reviews these regulating molecules and their potential in delineating the physiological and pathophysiological implications for BK channels.

### Regulation by Signaling Molecules or Endogenous Mediators

Physiologically, BK channel activity may be regulated *via* a wide variety of intracellular signaling molecules that bind to the cytoplasmic domain of the channel, including Mg^2+^, which depending on its concentration, can exert opposing effects in the activity of BK channels. While at physiological concentrations (0.5mM) and in the presence of relatively low Ca^2+^ levels, Mg^2+^ shifts the voltage-dependent opening of BK channels toward more hyperpolarized voltages, higher levels of this divalent cation (1–4mM) diminish BK channel unitary amplitude in a voltage-dependent manner (Zamoyski et al., 1989; Ferguson, 1991; McLarnon and Sawyer, 1993; Zhang et al., 1995, 2001; Morales et al., 1996; Shi and Cui, 2001; Shi et al., 2002; Xia et al., 2002).

There is also a growing literature on BK channel activity modulation by intracellular protons (Schubert et al., 2001; Schubert and Nelson, 2001; Avdonin et al., 2003; Brelidze and Magleby, 2004; Raingo et al., 2005; Park et al., 2007; Hou et al., 2009). Electrophysiological analysis using native smooth muscle cells from rat tail arteries revealed that while pH fluctuations (ranging 7.0–7.8) were unable to alter single-channel conductance or voltage-dependence of activation, the amplitude of intact BK channel currents were markedly decreased when lowering the pH from 7.2–6.8 (Schubert et al., 2001). In contrast, Dabertrand et al. (2012) elegantly demonstrated that brain acidosis induces the transformation of Ca^2+^ waves into Ca^2+^ sparks, leading to the activation of BK channels and subsequent dilation of brain parenchymal arterioles. The latter effect is due to the inherent ability of protons to bind to Ca^2+^ sensing residues located at the C-terminus of the BK channel. Specifically, intracellular protons target three residues [i.e., two Histidines (His) and, one Aspartate (Asp)] residing within the RCK1 domain of the BK channel (Avdonin et al., 2003).

Oxidative stress causes contrasting effects on BK channel function. Diverse studies in vascular smooth muscle have shown an increase in BK channel activity by oxidizing agents such as 5'5-dithiobis (2-nitrobenzonic acid, DNTB; Thuringer and Findlay, 1997), nicotinamide adenine dinucleotide (NAD) and glutathione sulfide (Lee et al., 1994), while other redox derivatives including dithiothreitol (DTT), β-mercaptoethanol, NADH or reduced glutathione (GSH) diminished BK currents (Thuringer and Findlay, 1997). Peroxynitrite, an oxidant produced by the near diffusion-controlled reaction between NO and superoxide ion, decreases BK channel open probability in cerebrovascular and coronary smooth muscle, leading to vessel constriction (Brzezinska et al., 2000; Liu et al., 2002). Furthermore, the inhibitory effect of peroxynitrite was reversible and thiol-dependent as the BK current amplitude was rescued by the antioxidant GSH. Acute or chronic deprivation of adequate oxygen supply (i.e., hypoxia) also influences the activity of BK channels with downstream effects on vascular tone. During short-term hypoxia, the brain vasculature dilates to increase cerebral blood flow, a mechanism of autoregulation which is thought to be partially mediated by BK channels (Gebremedhin et al., 1994). Similarly, Tao et al. (2015) demonstrated a stimulatory effect of chronic hypoxia on BK channel activity by increasing channel affinity for Ca^2+^ and shifting voltage channel activation to more hyperpolarized membrane potentials.

Heme, an essential cofactor involved in the redox-sensitive reaction of hemoproteins, directly binds to BK channel either in its oxidized or reduced state and drastically inhibits its activity (Tang et al., 2003). Further studies demonstrated that heme can impact the effect of allosteric activators of BK channels by possibly altering the architecture of the channel gating ring and thus, the voltage-sensing apparatus and the intrinsic stability of the open state of the channel (Horrigan et al., 2005). These findings suggest heme can function as a potent brake of BK channel activity, and it represents a clinically relevant agent with a potential cytoprotective role during conditions of “heme stress” or “excess of heme” such as trauma, ischemia or hypoxia (Gribkoff et al., 2001; Doré, 2002; Xu et al., 2002).

Carbon monoxide (CO) induces cerebral artery vasodilation through the activation of smooth muscle BK channels and thus increasing Ca^2+^ spark/STOC coupling. Specifically, CO reverses heme-induced BK channel inhibition by impairing the interaction between heme and its conserved binding domain (Jaggar et al., 2005). More detailed electrophysiology studies using inside-out, excised patch-clamp revealed that CO-induced BK channel activation is independent of the oxidation state of the gating ring of the channel and partly dependent on physiological levels of intracellular Ca^2+^ (Williams et al., 2008).

The action of various kinases including cAMP-dependent protein kinase (PKA), cGMP-dependent (PKG), and PKC directly regulates the apparent Ca^2+^- and/or voltage-sensitivity of BK channels, and thus, their physiological activity (Schubert and Nelson, 2001). Furthermore, BK channels are shown to contribute to the actions of many endogenous vasodilators [i.e., calcitonin-gene-related peptide (CGRP) or nitric oxide (NO)] that signal *via* adenylyl or guanylyl cyclase resulting in the elevation of cAMP or cGMP intracellular levels (Robertson et al., 1993; Miyoshi and Nakaya, 1995; Peng et al., 1996). In this context, several studies demonstrated the stimulatory influence of PKA and PKG phosphorylation on the kinetics of the BK channel, but not the conductance *per se*, causing a leftward voltage shift and increasing the open probability of the channel (Robertson et al., 1993; Minami et al., 1993a,b). In contrast, PKC phosphorylation directly attenuates BK currents in arterial smooth muscle (Schubert et al., 1999; Taguchi et al., 2000).

The activity of BK channels can be further modulated by the action of several membrane lipids (see Dopico and Bukiya, 2014 for a comprehensive review) including the minor but ubiquitous phospholipid component of cell membranes, phosphatidylinositol 4,5-bisphosphate (PIP_2_), the fundamental membrane lipid component cholesterol (Vaithianathan et al., 2008; Dopico et al., 2012; Tang et al., 2014; Tian et al., 2015; Dopico and Bukiya, 2017) and omega-3 fatty acids (Hoshi et al., 2013a,b). PIP_2_ directly stimulates vascular smooth muscle BK channels, contributing to vascular tone and blood flow control. Specifically, its negatively charged inositol head group interacts with a conserved motif in the cytoplasmic domain of the channel α subunit. The stimulatory effect of PIP_2_ is conferred by the accessory subunits that comprise the channel, potentiated by β_1_, which is abundantly expressed in smooth muscle, but not by β_4_ subunits. Consequently, pharmacological manipulation of endogenous PIP_2_ levels results in endothelium-independent dilation of cerebral resistance arteries, an effect that is blunted by selective BK channel blockers (Vaithianathan et al., 2008). Similarly, omega-3 fatty acids are known to potentiate BK channel activity in coronary artery smooth muscle and cause dilation of isolated coronary arteries (Lai et al., 2009; Wang et al., 2011; Hoshi et al., 2013a,b). Noteworthy, however, cholesterol down-regulates BK channels and its acute depletion using methyl-β-cyclodextrin potentiates channel activity (Dopico and Bukiya, 2014). However, a recent study using native cerebral artery smooth muscle cells revealed that cholesterol enrichment stimulated BK channels, and this effect was driven by increases in cell membrane levels of β_1_ subunits (Bukiya et al., 2021). In support to these findings, smooth muscle cells isolated from human coronary atherosclerotic plaque samples exhibited significantly higher channel activity than those obtained from coronary media segments (Wiecha et al., 1997). However, these findings should be interpreted with caution as cholesterol supplementation may directly modify the dynamic physical characteristics of the cell membrane, and consequently the conformation and function of the BK channel.

A long list of additional signaling molecules have been shown to influence BK channel activity including ethanol (Dopico et al., 1998; Bukiya et al., 2014a), paracrine mediators such as NO (Mistry and Garland, 1998) or adiponectin (Baylie et al., 2017); and hormones and circulating agents like angiotensin II (Zhang et al., 2014), leukotrienes (Bukiya et al., 2014b), ghrelin (Mladenov et al., 2008) or cannabinoids (Sade et al., 2006). Furthermore, BK channels are indirectly stimulated by a number of downstream second messengers resulting from the action of endogenous modulators such as adenosine and ATP, prostacyclin or CGRP (Cabell et al., 1994; Strøbaek et al., 1996a; Herzog et al., 2002; Tanaka et al., 2004).

### BK Channel Inhibitors and Blockers

Venom from scorpions represent a rich reservoir of bioactive peptides, some of which have robust BK channel inhibitory properties. These toxin peptides display high potency and selectivity, constituting powerful molecular tools for the biophysical characterization of BK channels and the development of BK channel pharmacology. Charybdotoxin (ChTX), a 37-amino-acid peptide obtained from the scorpion *Leiurus quinquestriatus hebraeus* (Gimenez-Gallego et al., 1988), was the first “BK channel blocker” reported. This potent peptide toxin binds electrostatically to the outer face of the BK channel and physically blocks its activity by interfering with K^+^ efflux through the ion conduction pathway. Despite its high-affinity, ChTX is known to block other subtypes of K^+^ channels including voltage-dependent K^+^ channels (K_v_1.2, K_v_1.3, and K_v_1.6) and intermediate-conductance calcium-activated K^+^ channels (Judge and Bever, 2006; Panyi et al., 2006), a property that results in its lack of selectivity and thus, prompts the requirement to use more selective BK channel blockers. The 37-amino-acid peptide Iberiotoxin (IbTX), a toxin purified from the African scorpion *Buthus tamulus*, shares extensive sequence homology (i.e., ~70%) with ChTX with an identical peptide backbone configuration, but exhibits more selectivity for BK channels as it does not inhibit other K^+^ channels apparently sensitive to ChTX (Galvez et al., 1990). The high selectivity of this toxin was determined by structural studies indicating that IbTX in fact binds to a different receptor on the external face of the BK channel which is allosterically coupled to the ChTX-binding site (Candia et al., 1992). This therefore, positions IbTX as a valuable pharmacological tool to study the structure and function of BK channels. In addition to ChTX and IbTX, a number of other toxin peptides have been purified from scorpion venom and similarly described as BK channel blockers with diverse and selective pharmacology, including Lq2 (Lucchesi et al., 1989), BmTx1/BmTx2 (Blanc et al., 1998), Lqh 15–1 (also called ChTx2; Marshall et al., 1994), and slotoxin (Garcia-Valdes et al., 2001). While all these peptide toxins are useful tools for experimentation, they do not display true potential as therapeutics given their inherent pharmaceutical disadvantages (e.g., rapid degradation, poor blood–brain permeability, ineffective orally active formulation) and poor reversibility.

A distinct group of highly selective and potent BK channel blockers include a series of non-peptide alkaloid molecules such as the fungal tremorgenic indole-diterpenes paxilline, penitrem A and lolitrem B, and the organic amines tetraethylammonium (TEA), verapamil and ketamine. While these alkaloids are capable of inhibiting BK channels in a highly specific fashion, their respective structures and mechanisms of action differ considerably (Kaczorowski et al., 1996; Nardi et al., 2003). Among them, tremorgenic mycotoxins, which are known to elicit a neurotoxic disorder in cattle called “ryegrass staggers” syndrome, are the most potent and selective non-peptide blockers of BK channels to date (Norris et al., 1980). In particular, paxilline has been the most extensively used in experimentation due to its apparent high-specificity and reversibility of action. This largely rigid molecule potently blocks BK channels at low nanomolar concentrations ([nM]) by interacting with binding sites residing on the α-subunit – distinct but allosterically coupled to those associated with ChTX (DeFarias et al., 1996; Sanchez and McManus, 1996). Additionally, a recent study from Zhou et al. (2020), identified a novel and highly specific site involved with paxilline-mediated inhibition that may represent a useful tool to further elucidate BK channel function as well as to design new modulators with promising clinical applications.

Quaternary amines such as TEA and its analogues belong to the group of organic amines able to block BK channels in a voltage-dependent manner but also a wide variety of other voltage-gated K^+^ channels, lacking therefore of high selectivity and applicability. In contrast to the toxin peptides – which only binds to the outer face of the BK channel – TEA blocks BK channels through either the internal or external side of the membrane, implying a complex mechanism of action. However, it exhibits different affinities depending on its site of action (external vs. internal). Specifically, BK channels are more sensitive to external TEA, and this particularity is attributed to a phenylalanine ring located near the mouth of the channel pore (Heginbotham and MacKinnon, 1992). This well-defined binding site is selective for TEA and it seems to act as a filter to differentiate the diverse TEA analogs by size. Given its ability to block BK channels, TEA has been suggested as a possible treatment to improve the persistent hypotension associated with septic shock. However, a study using a BK channel α subunit knockout mouse line demonstrated that BK channels are not a potential therapeutic target for sepsis-induced hypotension, suggesting therefore that the pressor effect of TEA may be attributed to other potassium channel species (O’Brien et al., 2011).

BK channel activity is also sensitive to other organic amines including verapamil and ketamine, quinoline derivatives such as quinine and quinidine, and imidazole derivatives (clotrimazole). The antihypertensive and antiarrhythmic agent verapamil and its analogues, are potent L-type Ca^2+^ channel blockers known to cause vasodilation and a decrease in arterial blood pressure. Verapamil is able to block BK channels (with an efficacy comparable to that reported for Ca^2+^ channels) by binding to a residue within the channel pore. The intravenous general anesthetic, ketamine is also reported to indirectly inhibit BK channels, and this effect is attenuated by increases in intracellular Ca^2+^ levels, suggesting that both ketamine and Ca^2+^ compete for the same binding site on the channel protein (Denson and Eaton, 1994). Among the quinoline derivatives, quinine and quinidine inhibit K^+^ efflux through BK channels, a blockade characterized by fast flickering of the channel between the open and closed states with a consequent reduction in open channel amplitude (Wong, 1989; Mancilla and Rojas, 1990). In addition, the imidazole antimycotic P450-inhibitor clotrimazole is also capable of diminishing the open probability of BK channels without affecting single-channel conductance (Wu et al., 1999).

Furthermore, a number of cations including H^+^, Na^+^, Cs^+^ and Ba^2+^ are also known to bind to the K^+^-conduction pathway and block single-channel (i.e., unitary) currents through BK channels, thus constituting vital experimental tools in characterizing their multi-ion pore conduction mechanism.

Direct blockade of BK channels may offer therapeutic benefit in certain pathologies. However, the use of the abovementioned peptide or non-peptide BK channels blockers in the clinical setting has been extremely limited due to their poor pharmaceutical features. Thus, a demand for more targeted and selective drugs still exists for meaningful pharmacotherapy strategies and improved patient outcomes. It is clear that only through dedicated research and development initiatives, we can expect a novel BK channel inhibitor compound to satisfy strict criteria for specific channel targeting and clinically acceptable pharmacokinetics/pharmacodynamics properties in humans.

### BK Channel Activators and Openers

Several synthetic and endogenous BK channels openers have been investigated at whole-cell and single channel levels using the patch-clamp technique in a diverse array of native vascular, non-vascular tissues from different animal species, and culture cell models. These small-molecule BK openers include the synthetic benzimidazoles NS004 and NS1619 (McKay et al., 1994; Lee et al., 1995a), the biaryl-amine flufenamic acid (Ottolia and Toro, 1994), the biarylurea NS1608 (Strøbaek et al., 1996b), the aryloxindol BMS-204352 (Gribkoff et al., 2001), the pyrrole derivative NS-8 (Tanaka et al., 2003), the indole-3-carboxylic acid esters CGS-7184 and CGS-7181 (Hu et al., 1997), and the natural modulator dihydrosoyasaponin-1 (Gribkoff et al., 1996). Among them, NS1619 has been widely studied as a potential therapeutic treatment for various conditions involving vascular and non-vascular smooth muscle such as shock-induced vascular hyporeactivity (Hu et al., 2014), pulmonary hypertension (Revermann et al., 2014), bladder hyperactivity (La Fuente et al., 2014), and erectile dysfunction (Gonzalez-Corrochano et al., 2013). However, the therapeutic perspective of NS1619 is limited given its relatively low potency and selectivity. A more selective BK channel opener, NS11021, has been reported to protect the heart against ischemia–reperfusion injury (Bentzen et al., 2009), enhance erectile responses in rodents (Kun et al., 2009) and reduce excitability and contractility of detrusor smooth muscle in the urinary bladder (Layne et al., 2010). Finally, the GoSlo-SR family constitute a group of anthraquinone analogues with higher potency than NS11021, which has been suggested as a useful starting template for the design of more tissue-specific BK openers (Roy et al., 2012).

While these BK channel openers would offer some limited clinical applications for conditions of neuronal and muscular hyperexcitability, they have not borne meaningful fruit for the pharmaceutical industry as therapeutic treatments. High on the list of potential reasons for difficulty to utilize such agents is that they may induce epilepsy and/or paroxysmal movement disorder(s). It appears that the abnormally increased BK channel activity may paradoxically lead to an enhancement in excitability in certain cases by triggering rapid depolarization of action potentials and therefore, contributing these pathological conditions (Du et al., 2005). Nonetheless, Cheney et al. (2001) demonstrated that the fluoro-oxindole BK channel opener BMS-204352 might be selectively beneficial for the treatment of experimental traumatic brain injury, in this case induced by lateral fluid percussion, as its administration significantly improved neurologic motor deficits and prevented the extent of regional cerebral edema at ~1–2weeks post-injury. This pharmacological agent was subsequently suggested as a promising therapeutic strategy for ischemic stroke as it was able to diminish neuronal excitability and excitatory transmitter release in a rodent model of stroke (Jensen, 2002).

## BK Channel Diversity

### Post-transcriptional Modifications

The pore-forming α-subunits of the mammalian BK channel are encoded by only one gene (*Slo1*; *KCNMA1*) which displays extensive alternative splicing of pre-messenger RNA. The powerful regulatory strategy of alternative splicing allows a large number of phenotypic splice variants to be generated from a single gene with high degree of diversity, particularly with respect to their physiological roles, tissue distribution, and biophysical features such as apparent sensitivity to calcium/voltage, unit conductance, activation/deactivation voltage range, and phosphorylation susceptibility by endogenous protein kinases or other intracellular signaling pathways (Tian et al., 2001; Chen et al., 2005; McCartney et al., 2005; Fodor and Aldrich, 2009). Alternative splicing also acts as a regulator of BK channel trafficking by finely tuning their cell surface expression according to certain physiological needs (Zarei et al., 2004; Singh et al., 2012). A variety of sites of alternative splicing within the α-subunits have been identified, and the intracellular C-terminal domain comprises the majority of them (Shipston, 2001). Using transcript scanning, Chen et al. (2005) analyzed the biophysical profile of five distinct splice variants resulting from alternative splicing at a single site – the mammalian site of splicing C2 residing in the C-terminal domain – and described the high variability among them in terms of functionality and biophysical properties. Thus, this widespread phenomenon represents a powerful mechanism to increase BK channel molecular heterogeneity and determine cellular excitability in a given tissue.

### Post-translational Modifications

BK channel activity is robustly regulated by an eclectic array of major post-translational processes including phosphorylation, palmitoylation, glycosylation and ubiquitination (for an extensive review see Shipston and Tian, 2016). For instance, BK channels are potently and reversibly controlled by PKA-mediated phosphorylation in neurons and smooth muscle cells (Lee et al., 1995b; Zhou et al., 2000). Several studies have identified various putative PKA-mediated phosphorylation C-terminal motifs including RQPS_899_ and the stress regulated exon (STREX), with remarkable properties contributing to promote either BK channel activation and inhibition, respectively (Tian et al., 2004). Interestingly, the cytosolic C-terminal of the STREX insert can also undergo palmitoylation of a conserved cysteine-rich domain, providing a conditional gate for BK channel regulation by PKA phosphorylation (Tian et al., 2008). Additional cysteine-enriched sites for palmitoylation have been identified – independent of and outside the STREX insert – within the intracellular linker between the S0 and S1 transmembrane domains with key roles in controlling BK channel cell-surface expression (Jeffries et al., 2010). This complex cross-talk between palmitoylation and phosphorylation explains the dramatic functional diversity of the BK channel among different cell types and tissues. N-linked glycosylation has been also reported to control BK channel stability, trafficking, and function. While direct evidence in the α subunits is sparse, β subunits have shown to be more susceptible to be N-glycosylated at two residues (Asn 53 and Asn 90 in the human β-4 subunit) in the large extracellular loop (Wallner et al., 1996; Jin et al., 2002). Finally, multiple sites in the C-terminal domain of the α subunits may be exposed to subsequent polyubiquitination which in turn results in BK channel accumulation in the endoplasmic reticulum. Accordingly, transgenic mouse models lacking the ubiquitination molecular machinery exhibit increased levels of BK channels at the cell surface and develop neuronal hyperexcitability and spontaneous epileptic seizures. These findings effectively suggest that this post-translational mechanism is critical to prevent this neurological disorder through BK channels (Liu et al., 2014).

### Association With Auxiliary Subunits

The association of the BK channel α-subunit with tissue-specific auxiliary subunits generates considerable functional channel diversity in several tissues and cell types of large mammals. Two main families of auxiliary proteins have been extensively characterized thus far, the regulatory β and γ subunits.

Among the β subunits, four different subtypes have been cloned (i.e., β1- β4) which share a similar architecture consisting of two transmembrane domains (i.e., TM1 and TM2) linked by a 100-amino acid extracellular loop, and short intracellular C- and N-terminals. Although to a different extent, each β subunits is generally able to impact the Ca^2+^ sensitivity, voltage dependence, and gating mechanisms of the BK channels they interact with, and thus, influence the cell membrane excitability in a tissue-specific manner (Brenner et al., 2000, 2005). Importantly, these auxiliary subunits may also alter the sensitivity of BK channels to regulatory molecules including hormones and lipids (King et al., 2006; Hoshi et al., 2013a; Martín et al., 2014). In regard to general tissue distribution, β-1 subunits are mainly found in vascular smooth muscle, urinary bladder and some areas of the brain, β-2 are highly expressed in chromaffin cells of the adrenal gland, pancreas, kidney and hippocampal neurons, β-3 is predominant in chromaffin cells, kidney, heart, liver and lung, and β-4 is almost exclusively expressed in the brain although it may be also found in smooth muscle (Contreras et al., 2013).

The family of auxiliary γ-subunits is equally composed of 4 distinct members (1γ- γ4), encoded by four different genes. The γ-subunit is made up of a single transmembrane segment, a large extracellular domain containing leucine-rich repeat proteins, and a short intracellular C-terminal domain. Specifically, these leucine-rich repeat proteins are critical in modifying the BK channel activation profile (Yan and Aldrich, 2010, 2012). More recently, a novel regulatory subunit termed LINGO1 has been discovered and constitutes the subject of ongoing studies. This protein, which shares a number of structural characteristics with γ_1-4_ subunits and has been associated with motor disorders and tremor such as Parkinson’s disease and essential tremor, was found to be in close association with BK channels and reduced BK channel activity in culture models and human cerebellar tissues (Dudem et al., 2020).

## Concluding Remarks

Many landmark studies have contributed to the understanding of BK channel structure and function. The reporting of the BK channel crystal structure had initially raised hopes about therapeutic potential, but difficulties generating a safe, reliable and specific pharmaceutical compound for therapy have been particularly problematic. The continued work to fully characterize the crystal structure(s) of the various BK channel accessory subunits may offer promise to provide alternative targets to modulate channel function and improve therapies. While such drugs and interventions may be some years away from clinical practice, efforts to study BK channel function using “laboratory-based” research compounds still provide important tools to further understand the various roles of BK channels in the context of cellular-, tissue-, and organ-specific studies.

## Author Contributions

MS and BDK wrote the manuscript, and designed the figures. Both authors contributed to the article and approved the submitted version.

## Conflict of Interest

The authors declare that the research was conducted in the absence of any commercial or financial relationships that could be construed as a potential conflict of interest.

## Publisher’s Note

All claims expressed in this article are solely those of the authors and do not necessarily represent those of their affiliated organizations, or those of the publisher, the editors and the reviewers. Any product that may be evaluated in this article, or claim that may be made by its manufacturer, is not guaranteed or endorsed by the publisher.
